# Assessing Risks to Non-Target Species during Poison Baiting Programs for Feral Cats

**DOI:** 10.1371/journal.pone.0107788

**Published:** 2014-09-17

**Authors:** Tony Buckmaster, Christopher R. Dickman, Michael J. Johnston

**Affiliations:** 1 School of Biological Sciences, University of Sydney, New South Wales, Australia; 2 Invasive Animals Cooperative Research Centre, University of Canberra, Australian Capital Territory, Australia; 3 Department of Parks and Wildlife, Wanneroo, Western Australia, Australia; University of Kent, United Kingdom

## Abstract

Poison baiting is used frequently to reduce the impacts of pest species of mammals on agricultural and biodiversity interests. However, baiting may not be appropriate if non-target species are at risk of poisoning. Here we use a desktop decision tree approach to assess the risks to non-target vertebrate species in Australia that arise from using poison baits developed to control feral house cats (*Felis catus*). These baits are presented in the form of sausages with toxicant implanted in the bait medium within an acid-soluble polymer capsule (hard shell delivery vehicle, or HSDV) that disintegrates after ingestion. Using criteria based on body size, diet and feeding behaviour, we assessed 221 of Australia's 3,769 native vertebrate species as likely to consume cat-baits, with 47 of these likely to ingest implanted HSDVs too. Carnivorous marsupials were judged most likely to consume both the baits and HSDVs, with some large-bodied and ground-active birds and reptiles also consuming them. If criteria were relaxed, a further 269 species were assessed as possibly able to consume baits and 343 as possibly able to consume HSDVs; most of these consumers were birds. One threatened species, the Tasmanian devil (*Sarcophilus harrisii*) was judged as definitely able to consume baits with implanted HSDVs, whereas five threatened species of birds and 21 species of threatened mammals were rated as possible consumers. Amphibia were not considered to be at risk. We conclude that most species of native Australian vertebrates would not consume surface-laid baits during feral cat control programs, and that significantly fewer would be exposed to poisoning if HSDVs were employed. However, risks to susceptible species should be quantified in field or pen trials prior to the implementation of a control program, and minimized further by applying baits at times and in places where non-target species have little access.

## Introduction

Invasive mammalian predators pose problems for agricultural production and especially for the conservation of biodiversity in many parts of the world [1]. Prey species that have evolved on islands in the absence of mammalian predators often are highly susceptible to the arrival of new invasive forms [2], but even prey inhabiting large mainland areas can be driven to extinction if a new predator ‘archetype’ is introduced [3], [4]. In Australia, small native mammals and birds have experienced depredation by carnivorous marsupials (0.5–10 kg) since Miocene times to the present [5], yet many species in these prey groups have suffered serious population declines or extinction following the arrival of two novel predators, the red fox (*Vulpes vulpes*) and feral house cat (*Felis catus*) [6]–[9] in the wake of European colonization. Meta-analyses confirm that invasive alien predators have more strongly negative effects on prey species than do native predators, although the effects of foxes and cats in Australia generally are more pronounced than the impacts of invasive predators elsewhere in the world [10], [11].

In Australia, the control of invasive predators for the conservation of biodiversity and protection of primary production assets is conducted principally through the use of poison baits [12]. However, a key consideration when using a toxicant for the control of any species is the likelihood that non-target species will take the bait [13], [14]. Consumption of baits by non-target species can have unintended consequences ranging from incapacitation to death [15]–[17], to limiting the efficacy of the control program through monopolization of baits [18]. Baits also are used for the delivery of oral vaccines for disease control [19], [20] and potentially for the dissemination of immuno-contraceptives [21]. In line with community expectations [22], the development of new baits should be as target-specific as possible.

Because of its acute toxicity to most mammals, the poison sodium monofluoroacetate (1080) is used widely to control mammalian pests; in Australia, it is the only toxicant that is currently registered and commercially available for predator control [23]–[26]. Many native mammal species in western and north-western Australia are relatively tolerant to this toxicant as they coevolved with endemic plant species that contain naturally occurring fluoroacetate compounds [27]–[29]. However, mammals in eastern Australia are less tolerant and more susceptible to 1080 toxicosis than their western counterparts and, as a result, 1080 baits laid in eastern Australia are generally buried to minimize the potential for non-target species to access them [28], [30]. Bait burial provides little obstacle for canid species as their acute olfactory senses allow them to detect buried baits which are then excavated and consumed [31]. Baits buried at 15 cm are excavated readily by foxes but less so by native species [32], and can be very effective at reducing the activity and numbers of foxes over large areas [33], [34].

Feral cats, by contrast, do not possess the same acute olfactory senses as canids and rarely locate and excavate buried baits [35], preferring live prey over carrion or dried meat baits [36]. Therefore baits intended for feral cats must be surface-laid, attractive and palatable [35], [37]. However, surface application increases the potential for non-target species to encounter the baits and consume lethal amounts of toxicant [14].

To improve the efficiency of using poison baits for feral cats while reducing risks to non-target species, three sets of trials have been initiated in Australia. First, research has focused on developing a bait medium that is attractive and palatable to feral cats. The most effective medium to date, Eradicat, is manufactured by the Department of Parks and Wildlife in Western Australia (Patent number AU 781829). It consists of moist kangaroo meat, chicken fat, digests and flavour enhancers and is presented on the soil surface in the form of chipolata-style sausages ∼15 g in weight and 10 cm in length [17], [35]. In Western Australia, Eradicat baits are injected directly with 4.5 mg of 1080 and, depending on bait density and environmental conditions, can reduce cat activity by >80% [35], [37]. Despite the efficacy of these baits, however, the manner of their poison delivery means that they are not suitable for use in areas with 1080-susceptible non-target fauna.

Second, para-aminopropiophenone (PAPP) has been developed as an alternative toxicant for 1080 in at least some control applications [26], [38]–[40] as its adoption will lead to improved safety for the humans preparing baits along with a more humane toxicosis in the feral cat based on the observable symptoms. For many species, LD_50_ rates–the amount of toxicant needed to kill 50% of the sample group–for PAPP are far higher than those for 1080. For example, the LD_50_ for PAPP for *F. catus* is 5.56 mg kg^−1^ [41] while for 1080 it is 0.28 mg kg^−1^ [42] to 0.4 mg kg^−1^ [43]. Having a lower toxicity, non-target species must consume more toxicant to obtain a lethal dose than would be required if using 1080. While the LD_50_ in cats for PAPP is an order of magnitude higher than that for 1080, sufficient toxicant is contained within a single bait. As a result, only a single bait needs to be consumed by cat to result in death. Multiple bait consumption has been observed in field efficacy studies and has prompted consideration about optimising the bait density and procedure for aerial application [44].

Third, encapsulation of toxicant (of PAPP initially, but more recently of 1080) within bait media is being trialled as a means of minimizing the exposure of non-target species to the toxicant [17], [45], [46]. Here, toxicant is sealed within a hard, acid-soluble polymer capsule known as a hard shelled delivery vehicle (HSDV), and the HSDV is then inserted into the bait prior to it being laid. The HSDV exploits differential feeding behaviour between the target and many non-target species to minimize exposure of the non-target species to the toxicant [45]. Many animals gnaw at baits rather than consuming them with a single, or several, large bites [17]. Given this feeding behaviour, native animals are more likely to reject or spit out the HSDV rather than consume it. [17], [45]. However, if consumed the HSDV dissolves in the stomach and releases the toxicant.

Statutory authorities that approve and register new baits or methods of bait presentation require extensive field and pen trials to determine which non-target species will take baits [47], [48]. This information will be crucial in any future feral cat-control programs that use surface-laid baits containing PAPP [49]. Here, we use a desktop risk assessment approach to identify non-target species that are likely to take such baits based on key aspects of their biology, and thus reduce the number of species that need to be subjected to field or pen-trial tests. We focus on native Australian species owing to the imperative to reduce ongoing impacts on the many species that feral cats threaten [6], [49], but emphasize that our methodology is generic and can be applied to any situation where similar approaches to feral cat-control are being considered. We first examine the likelihood that potential prey species will eat bait media presented to feral cats, and then assess the likelihood that they may ingest an HSDV with toxicant that has been embedded in the bait. We evaluate all Australian species, but also highlight those listed as vulnerable or endangered as these frequently are the species that poison baiting programs seek to protect. Based on the results, we provide suggestions to managers about how best to minimize the possibility of poisoning non-target species during campaigns to control feral cats. We stress that the process we have used is not a replacement for carefully designed and rigorously undertaken pen and field trials, but rather is an initial step in the process of managing and minimizing non-target access to toxicants during a baiting program. The process should also not diminish the requirements for selecting target-specific bait and appropriate bait placement.

We define feral cats as cats that live and reproduce in the wild and survive by hunting or scavenging, with none of their needs satisfied intentionally by humans [50].

## Methods

### Study area and species

We evaluated vertebrate species that occur in all parts of mainland Australia, Tasmania and offshore islands, and also species that occur on Australian external territories. These include Ashmore, Cartier, Christmas, Cocos (Keeling), Coral Sea, Lord Howe, and Norfolk Islands in the Indian and Pacific Oceans, as well as the sub-Antarctic Heard, McDonald and Macquarie Islands.

### Bait and HSDV capsule

The bait medium we chose for this analysis was based on Eradicat, but without the injected 1080 poison and with buffering to pH 7.5 to ensure stability of the HSDV within the bait. The HSDV capsule we selected is a proprietary product manufactured by Scientec Research Pty Ltd (Melbourne, Victoria, Australia) and designed specifically to encapsulate PAPP toxicant formulation (Provisional Patent No. 200890357). This HSDV measures 6 mm in diameter and 8 mm in length. It is formulated to dissolve swiftly in stomach acid [46], [51]. Used together, the bait medium with implanted HSDV is being commercialized as Curiosity bait; preliminary trials show that it can be attractive to feral cats and effective in reducing their numbers [26], [46], although variable efficacy occurs in relation to other factors, such as alternate food availability [52].

### Lists of potential non-target species

Comprehensive lists of all Australian terrestrial vertebrate species and subspecies were obtained from the Australian Biological Resources Study (ABRS) within the Department of the Environment, Water, Heritage and the Arts (now Department of the Environment) for the four taxonomic classes analyzed: Amphibia [53], Aves [54], Mammalia [55], and Reptilia [56].These lists are accessible at (http://www.environment.gov.au/biodiversity/abrs/index.html) and are assumed to be comprehensive and to provide the most current scientific name for each species and subspecies. The lists include accidental and occasional visitors to the Australian mainland and offshore islands. The species data files were downloaded directly from the ABRS website as CSV files and then converted into Excel spreadsheets. Superfluous data (for example, species synonyms, details of amendments to species' common and scientific names and historical nomenclature) were removed. The remaining data included for each species: common name, current scientific name and recognized subspecies. All assessments were undertaken using these modified lists.

To assess the likelihood of native species consuming baits, with or without HSDVs, we gathered information on animal size, habits, diet, conservation status and other aspects from a wide variety of field guides, checklists and other published sources (see below). Due to the continual and ongoing revision of the taxonomy of Australian vertebrates [e.g. 57,58], we took care to ensure that all species referred to in the literature were the same as those on the ABRS CSV files. In several instances the ABRS database listed animals that were not described in any guides or checklists; for these taxa we searched recent literature to obtain information on their body size and other biological characteristics. Generally such discrepancies arose when a previously described species had been reclassified into multiple new species for which no descriptions were available outside the primary source. Common names for species were taken from the ABRS data files. We undertook the final analysis on species lists as they stood with the ABRS as of 9 March 2009; any subsequent taxonomic revisions of Australian vertebrates have not been included.

### Potential for non-target species to take baits or HSDVs

As we did not carry out any field or pen-trial testing of animals for this analysis, we assessed the bait-take potential for each species using decision tree analysis [59], with assessment criteria listed in Appendixes S1and S2. The criteria were developed to assess the potential for each species to consume either the chipolata-style bait medium and/or the implanted HSDV, and included diet, feeding behaviour and animal size.

In general, we assumed that mammals would be likely to encounter and consume surface-laid chipolata-style bait media if they were terrestrial and broadly insectivorous or carnivorous, whereas classes of other terrestrial vertebrates with similar diets would likely consume baits only if they were large enough to do so (Appendix S1). This size-based difference recognizes the ability of small mammals to gnaw large food items; other small vertebrates lack the teeth to do this. We assumed that omnivores that eat mostly green plants or fungi (e.g. potoroids) could possibly take baits, but that herbivores, species specializing on other plant products such as nectar or seeds, and species that feed on the wing and in or above water, would not be at risk of either taking baits or of consuming dead animals that had eaten baits (Appendix S1).

In identifying animals that might consume HSDVs, we included only those species that were assessed in the decision process as being at least possibly able to consume the bait media. For these species, we assumed that a broadly carnivorous, insectivorous or generalist diet would be likely associated with HSDV consumption, but only in animals that were large enough to swallow a HSDV capsule (Appendix S2).

We obtained relevant information for the assessment criteria from morphometric data, dietary and habitat preferences listed in species guides for mammals [60], amphibians and reptiles [61], [62], and birds [63]. We used maximum weights (g) for mammals or, where available, maxima for the usual weight range. For birds we used total length (cm) inclusive of the tail, for snakes and turtles the combined body plus tail length, and for other reptiles and amphibians we used snout – vent length (cm). Information on diet and habitat use was available for most mammal species, but for many other vertebrates it was given only at the family level; hence our assessments also were undertaken at this level. After data compilation, we checked our results against those of the few field or pen-trial studies in which species have been presented with baits and HSDVs (e.g. [17], [45]) to confirm the reliability of our assessment approach. We also contacted researchers with as-yet unpublished results from field or pen-trial studies on other species to provide a further check on the accuracy of our assessments. Each species' national conservation status was based on listings of threatened (vulnerable or endangered) taxa from Clayton et al. [64], as defined by the Commonwealth *Environment Protection and Biodiversity Conservation Act 1999*.

### Analysis

Results are presented largely as tallies of species assessed as able, or possibly able, to consume sausage-style baits, and tallies of species at further risk of ingesting HSDVs. We used chi-squared tests to compare the numbers of species in these two categories, with Yates' correction for comparisons with one degree of freedom.

## Results

In total 3,769 native Australian vertebrate species and subspecies were evaluated in this review, and 490 were determined as able to consume, or possibly able to consume, the Curiosity bait media if deployed on the ground surface. Of these, only 47 species were assessed as able to consume HSDVs within the bait media, although consumption was judged to be possible for a further 343 species (Table 1) based on the criteria in Appendixes S1and S2. Electronic supplementary material contains a complete list of all species used in this analysis and the complete results listed by taxonomic class.

**Table 1:** Numbers of native Australian vertebrate species in four taxonomic classes evaluated in this analysis and the numbers within each class assessed as able to consume, or possibly able to consume, sausage-style bait media and toxicant encapsulated in a hard shelled delivery vehicle (HSDV).

Group	No. assessed	Bait		HSDV	
		Will consume	Possibly consume	Will consume	Possibly consume
Mammalia	582	157 (27)	20 (3.4)	21(3.6)	69 (11.8)
Aves	1,872	24 (1.2)	239 (12.8)	12 (0.6)	239 (12.8)
Reptilia	1,086	40 (3.7)	10 (0.9)	14 (1.3)	35 (3.2)
Amphibia	229	0 (0)	0 (0)	0 (0)	0 (0)
Total	3,769	221 (5.9)	269 (7.1)	47 (1.3)	343 (9.1)

Assessments are based on decision criteria in Appendixes S1and S2, The value in brackets is the percent of the total number assessed for each group.

Within classes, mammals generally were assessed as best able to consume baits and HSDVs, but more birds were judged as possible consumers. Dasyurid and peramelid marsupials and murid rodents showed strongest potential to consume baits, but only the larger members of these families appeared likely to consume HSDVs too. Large, ground-active birds such as ardeids and megapodes were the likeliest consumers of both baits and HSDVs, with generalist foragers such as corvids being represented strongly also. Among the Reptilia, some snakes, varanids and large skinks were assessed as being able to consume baits or HSDVs, but no Amphibia were assessed as potential consumers (Table 1).

Overall, far fewer species were judged able to consume HSDVs within baits than the chipolata-style bait media alone (χ^2^_3_ = 140.7, *P*<0.001). This effect was strongest for mammals, which showed a >7-fold reduction in the number of species able to consume baits compared with HSDVs (χ^2^_1_ = 913.7, *P*<0.001), but equivalent 2–3-fold reductions were significant also for birds (χ^2^_1_ = 12.1, *P*<0.001) and reptiles (χ^2^_1_ = 48.9, *P*<0.001).

The Tasmanian devil (*Sarcophilus harrisii*) was the only threatened (endangered) species assessed as definitely likely to reliably consume chipolata-style baits with implanted HSDVs. However, a further 21 threatened species were possible consumers of HSDVs (Table 2) based on their diet and feeding behaviour (Appendixes S1and S2). Five of these were birds, the others dasyurid, peramelid and potoroid marsupials.

**Table 2:** Threatened species of native Australian vertebrates assessed as being possibly able to consume baits containing hard shelled delivery vehicles during ground surface baiting campaigns for feral cats.

Common name	Scientific name	Status
Southern cassowary	*Casuarius casuarius*	E
Malleefowl	*Leipoa ocellata*	V
Antarctic tern subsp. bethunei	*Sterna vittata bethunei*	E
Antarctic tern subsp. vittata	*Sterna vittata vittata*	V
Pied currawong subsp. crissalis	*Strepera graculina crissalis*	V
Brush-tailed bettong subsp. ogilbyi	*Bettongia penicillata ogilbyi*	E
Crest-tailed mulgara	*Dasycercus cristicauda*	V
Ampurta	*Dasycercus hillieri*	E
Kowari	*Dasyuroides byrnei*	V
Western quoll (Chuditch)	*Dasyurus geoffroii*	V
Northern quoll	*Dasyurus hallucatus*	E
Spotted-tailed quoll	*Dasyurus maculatus gracilis*	E
Spotted-tailed quoll	*Dasyurus maculatus maculatus*	E/V
Golden bandicoot subsp. auratus	*Isoodon auratus auratus*	V
Golden bandicoot subsp. barrowensis	*Isoodon auratus barrowensis*	V
Dibbler	*Parantechinus apicalis*	E
Eastern barred bandicoot unnamed subsp.	*Perameles gunnii* subsp. (Victoria)	E
Eastern barred bandicoot subsp. gunnii	*Perameles gunnii gunnii*	V
Red-tailed phascogale	*Phascogale calura*	E
Northern brush-tailed phascogale	*Phascogale tapoatafa pirata*	V
Tasmanian devil	*Sarcophilus harrisii*	E
Julia Creek dunnart	*Sminthopsis douglasi*	E

Status listings (E =  endangered, V =  vulnerable) were obtained from Clayton et al. [61], as defined under the *Commonwealth Environment Protection and Biodiversity Conservation Act 1999*.

## Discussion

Our analyses reveal that most native Australian vertebrates would not be exposed to non-target poisoning if surface-laid baits are used during programs to control feral cat populations. Significantly fewer non-target species would access toxicants that are enclosed within HSDVs than would occur if the toxicant was injected directly into the bait media, as is the current practice with Eradicat bait. In pen trials, Hetherington et al. [17] confirmed that use of HSDVs would reduce the potential impacts of poisoning campaigns on western quolls (*Dasyurus geoffroii*), brush-tailed bettongs (*Bettongia penicillata*) and southern brown bandicoots (*Isoodon obesulus*), while Marks *et al.* [45] found that HSDVs reduced the ability of northern quolls (*Dasyurus hallucatus*) to access toxicants within feral cat baits. More recent studies with HSDVs containing rhodamine-b dye have helped to confirm that these devices are consumed by few non-target species [65].

Although baits with HSDVs could be expected to reduce non-target mortality, there were nonetheless large differences in the likelihood of bait and HSDV uptake among vertebrate classes. Mammals, particularly carnivorous marsupials, were judged to be the most susceptible group, followed by reptiles and birds, although more birds were rated as being possibly able to take baits and HSDVs than other vertebrates. Amphibians were considered too small to be able to swallow baits, and hence would not be at risk during any campaigns using the Curiosity bait. The higher risk of carnivorous marsupials to baits is not surprising; these animals are ecologically most similar to the feral cat, for which the Curiosity bait has been designed. Most prefer to hunt live prey, but many also have been recorded to scavenge and will readily eat moist minced meat in captivity [66], [67]. However, far fewer species were judged likely to consume HSDVs compared with the bait media, and only *Sarcophilus harrisii* among the threatened taxa was rated able to take an HSDV with any degree of certainty. Although 16 other species of threatened marsupials were assessed as possibly able to ingest HSDVs (Table 2), for most of these the likelihood of actual consumption probably is very low. *Parantechinus apicalis*, for example, will readily consume the carcass of a 15 g house mouse (*Mus musculus*) in the field, but not the hard bones of the jaw or limbs that most resemble HSDVs in size and hardness [68]. Relatively few birds and reptiles were judged as able to consume HSDVs, and all of these were large terrestrial species capable of swallowing baits whole. Of the threatened bird taxa possibly able to consume HSDVs (Table 2), risks are likely measurable for the ground-fossicking *Casuarius casuarius* and *Leipoa ocellata* but negligible for the remaining three taxa. These latter species occupy islands where cats do not occur [69] or have been recently eradicated [70].

Because of the desktop nature of our analyses, there were several questions that we did not address. First, we did not consider the possibility of unintentional consumption of bait media in our analyses. For example, a large obligate herbivore would not actively seek out and consume a meat-based bait, but may unintentionally consume one while foraging. To quantify the probability of such occurrences more precisely would require extensive field or pen testing, and was beyond the scope of this analysis. However, such events would likely be very rare and certainly not lead to population level declines through unintended poisoning of non-target species. Second, we did not address whether bait density, bait placement or habitat might affect the likelihood of species encountering and consuming baits, even though these aspects are important in field programs [37]. Because we evaluated the likelihood of bait consumption only when baits had been encountered, it is possible that our assessments identified more species as being at potential risk than would be the case in the field. However, such bias is arguably warranted from a precautionary perspective [71]. Third, we did not assess the possibility of toxicants leaking into the bait media after gnawing or degradation of the HSDV capsule, resulting in the encapsulated toxicant becoming available to non-target species. Although this is possible, it is unlikely to be a persistent problem; the manufacturing process is being modified to harden the HSDV material (M. O'Donoghue, Scientec Research Pty Ltd, personal communication). As HSDVs alone are unattractive and have no energetic or nutritional value, they are not likely to pose any threat to non-target species if they are removed or ejected from bait media by other foragers.

By examining the behaviour and feeding patterns of possible non-target species, it should be possible to minimize the potential for bait-take by these species still further. For example, many carnivorous marsupials occupy relatively small and stable ranges during spring as invertebrate food resources become active [72], suggesting that they would be less likely to encounter and eat baits if set during this season. Large varanid lizards, which have a high potential to consume Curiosity bait media and HSDVs, similarly are less active in winter than in warmer months, and would likely be little affected by a baiting campaign in late winter in south-eastern Australian states. As feral cats also are likely to be food stressed in winter and more likely to take baits at this time [35], [73], the winter period thus could provide a window of opportunity to minimize bait-take by non-target species and maximize uptake by feral cats.

In addition to optimizing timing, non-target bait-take could be minimized by careful placement of baits. For example, many non-target mammal species that are likely to consume baits are small (<200 g) and terrestrial, and unlikely to access baits placed above ground. Algar and Brazell [18] devised a simple gantry device to suspend baits 30–40 cm above ground and out of the reach of black rats (*Rattus rattus*), wild chickens (*Gallus domesticus*) and land crabs (*Cardisoma carnifex*) on Christmas Island. This device minimized bait-take by rats, birds and crabs, but kept the baits available for feral cats [18], [46]. Modifications might be possible to heighten the gantry to place baits out of reach of large murids and peramelid marsupials, or to place it in different microhabitats where particular non-target species are less active [18].

### Management implications

Our results suggest that many of the risks to non-target species that arise using surface-laid baits (including aerial applications) for invasive pest animals can be reduced, but not negated entirely, by encapsulating toxicant within HSDVs. We suggest further that successful encapsulation may even allow other compounds such as alternative toxicants, vaccines or other drugs that usually cannot be used in surface-laid baits to be considered for use in future. More generally, we have shown that a decision tree process can provide a simple, transparent and repeatable assessment method for identifying species that are likely to take baits and/or HSDVs. This approach also allows for the incorporation of new data when they become available, and could be adapted for use with other bait types or other invasive pest species. We emphasize that this method of assessment should not be seen as a replacement for rigorous bait-take trials in pens or the field. However, we suggest that it can provide the initial steps in a considered process of managing and minimizing risks to non-target species that might otherwise be harmed in control programs for invasive or other pest species.

## Supporting Information

Appendix S1 **Criteria used for assessing the potential to consume chipolata-style (Curiosity) baits designed for feral cats.** Assessment is modified by each subsequent level. For example, a carnivore that feeds predominantly at sea will be assessed as having no potential to consume a bait. When a “No” assessment is made, decision analysis for that animal ceases.
(DOCX)

Appendix S2 **Criteria used for assessing the potential to consume toxins encapsulated in hard shelled delivery vehicles (HSDVs) implanted within chipolata-style bait media intended for feral cats.** Assessment is modified by the subsequent level. For example, a large carnivore that has demonstrated complete HSDV rejection in field or pen studies will be assessed as having no potential to consume the HSDV. When a “No” assessment is made, decision analysis for that animal ceases.
(DOCX)
