# Supplementary material for: Assessing Risks to Non-Target Species during Poison Baiting Programs for Feral Cats
Source: PLoS One. 2014 Sep 17;9(9):e107788. doi: 10.1371/journal.pone.0107788 (PMC4168141; doi:10.1371/journal.pone.0107788)
Supplement: Appendix S1 — Criteria used for assessing the potential to consume chipolata-style (Curiosity) baits designed for feral cats. Assessment is modified by each subsequent level. For example, a carnivore that feeds predominantly at sea will be assessed as having no potential to consume a bait. When a “No” assessment is made, decision analysis for that animal ceases. (DOCX) [file pone.0107788.s001.docx]

**Appendix S1**. **Criteria used for assessing the potential to consume chipolata-style (Curiosity^®^) baits designed for feral cats.** Assessment is modified by each subsequent level. For example, a carnivore that feeds predominantly at sea will be assessed as having no potential to consume a bait. When a “No” assessment is made, decision analysis for that animal ceases.

| **Criteria** | **Assessment of potential** |
| --- | --- |
| **A. Diet** |  |
| Carnivore or scavenger, including scavenging omnivores | Yes – unless otherwise shown by lab or field testing |
| Insectivore, including those consuming other invertebrates or occasional small vertebrates | Yes – unless otherwise shown by lab or field testing |
| Omnivore, including those consuming mostly plant materials | Possible – unless otherwise shown by lab or field testing |
| Hypogeal fungivore | Possible – unless otherwise shown by lab or field testing |
| Herbivore or frugivore or exudate feeders, including specialist or obligate feeders | No |
| Volant insectivores | No |
| Granivorous birds | No |
| **B. Feeding behaviour** |  |
| Feeds predominantly at sea or on the wing | No |
| Specialist ant feeder | No |
|  |  |
| Unlikely to recognise bait as food source; e.g., cursorial carnivores with minimal scavenging | No |
| **C. Size** |  |
| Birds or amphibians or reptiles too small to consume bait^a^ | No |

^a^For this category, birds < 26 cm total length, amphibians < 15 cm and reptiles < 30 cm snout – vent length or 50 cm total length were assumed to be too small to eat baits.
