# Supplementary material for: Assessing Risks to Non-Target Species during Poison Baiting Programs for Feral Cats
Source: PLoS One. 2014 Sep 17;9(9):e107788. doi: 10.1371/journal.pone.0107788 (PMC4168141; doi:10.1371/journal.pone.0107788)
Supplement: Appendix S2 — Criteria used for assessing the potential to consume toxins encapsulated in hard shelled delivery vehicles (HSDVs) implanted within chipolata-style bait media intended for feral cats. Assessment is modified by the subsequent level. For example, a large carnivore that has demonstrated complete HSDV rejection in field or pen studies will be assessed as having no potential to consume the HSDV. When a “No” assessment is made, decision analysis for that animal ceases. (DOCX) [file pone.0107788.s002.docx]

**Appendix S2.** **Criteria used for assessing the potential to consume toxins encapsulated in hard shelled delivery vehicles (HSDVs) implanted within chipolata-style bait media intended for feral cats.** Assessment is modified by the subsequent level. For example, a large carnivore that has demonstrated complete HSDV rejection in field or pen studies will be assessed as having no potential to consume the HSDV. When a “No” assessment is made, decision analysis for that animal ceases.

| **Criteria** | **Assessment of potential** |
| --- | --- |
| **A. Bait consumption** |  |
| Where potential for bait consumption = No | No |
| **B. Size and diet** |  |
| Large eutherian carnivore^a^ | Yes |
| Carnivore or insectivore larger than smallest known to consume HSDV (determination based on body size and feeding habits) | Possible or Yes |
| Carnivore or insectivore smaller than smallest known to consume HSDV (determination based on body size and feeding habits) | Possible or No |
| Omnivore larger than smallest mammal known to consume HSDV with similar feeding habits to those known to consume HSDVs | Individual determination |
| Omnivore smaller than smallest known to consume HSDV | No |
| **C. Field and pen studies** |  |
| Complete HSDV rejection confirmed by lab or field experiment | No |
| Predominant HSDV rejection demonstrated by lab or field experiment | Possible, but unlikely with data shown |
| Predominant HSDV consumption demonstrated by lab or field experiments | Yes |

^a^For this section a large eutherian carnivore is defined as a cat (*Felis catus*) or larger.
